# Research Progress on the Application of Upconversion Nanoparticles in Heavy Metal Detection in Foodstuff

**DOI:** 10.3390/foods14234144

**Published:** 2025-12-03

**Authors:** Zhiqiang Chen, Kangyao Zhang, Ye He

**Affiliations:** 1 School of Food Engineering, Zhangzhou Institute of Technology, Zhangzhou 363000, China; 2 School of Advanced Manufacturing, Fuzhou University, Quanzhou 362251, China; 3 College of Public Health, Fujian Medical University, Fuzhou 350108, China

**Keywords:** upconversion nanoparticles (UCNPs), heavy metals, food safety, detection technologies

## Abstract

Heavy metal contamination in foodstuff poses a serious threat to food safety and human health; therefore, the development of toxic heavy metal detection methods is crucial. However, lots of these methods, based on traditional nanomaterials, have unavoidable limitations, such as high instrument cost, complicated operation procedures, or a long analysis time, which restrict their wide application in heavy metal detection. This review aims to conduct a systematic overview of major analytical methods using novel upconversion nanoparticles (UCNPs) for assessing heavy metal ions in complex food matrices in the context of food safety and show their potential application prospects when combined with big data and artificial intelligence. Due to their unique optical properties, good bio-compatibility, and tunable interfacial chemistry, UCNPs have shown significant detection advantages in the field of food heavy metal analysis. The review summarizes the progress of the application of UCNPs in heavy metal detection in food. Despite the development of new technologies such as artificial intelligence, and the continuous optimization and improvement of its own design, the wide application of UCNPs in food safety detection still has great potential for further development.

## 1. Introduction

Heavy metals, such as lead, mercury, cadmium, arsenic, and chromium, are metallic or metalloid elements with densities exceeding 4 ± 1 g/cm^3^, comprising approximately 45 naturally occurring elements [1,2]. Human activities, including industrial emissions, agricultural inputs (fertilizers and pesticides), mining, and waste disposal, can increase the concentration of heavy metals in the environment, leading to environmental degradation when this exceeds the environmental carrying capacity, resulting in heavy metal pollution. These metals enter soil and water through sewage and atmospheric deposition, accumulating in the food chain via bioconcentration. Consequently, heavy metals levels in foods like rice, fish, shellfish, and animal offal often exceed the safe limits. Chronic consumption poses significant health risks, including neurotoxicity, liver and kidney damage, and carcinogenic and teratogenic effects, with pronounced impacts on child development [3,4,5,6]. Unlike other pollutants, heavy metals are cumulative, irreversible, non-degradable, and biomagnified [7], posing persistent environmental challenges.

Heavy metal contamination in food poses a serious threat to food safety and human health, so the development of effective heavy metal detection methods is crucial. Traditional heavy metal detection methods include atomic emission spectrometry, atomic absorption spectrometry, atomic fluorescence spectrometry, inductively coupled plasma mass spectrometry, inductively coupled plasma atomic emission spectrometry, and surface-enhanced Raman spectroscopy [8]. However, these methods have unavoidable limitations, such as high instrument costs, complicated operation procedures, or long analysis times, which restrict their wide application in rapid food detection.

To solve these problems, researchers have developed various heavy metal detection sensors, such as electrochemical sensors, spectroscopic biosensors, and colorimetric biosensors, using nanomaterials including metallic nanomaterials, inorganic nonmetallic nanomaterials, organic nanomaterials, and composite nanomaterials [9]. The application of the above nanomaterials in sensors not only improves the sensitivity and selectivity of heavy metal detection, but also provides the sensors with the advantages of low cost, easy operation, and rapid detection, and therefore has received focused attention.

Fluorescence sensing technology based on fluorescent sensors is a novel analytical method that utilizes fluorescent molecules in fluorescent nanomaterials to specifically bind with the target molecules to trigger fluorescence quenching or enhancement effects. Its application in heavy metal detection in food products is mainly based on the design of fluorescent probes and the mechanism of photophysical signal conversion to enhance the sensitivity and anti-interference ability of heavy metal ions. However, traditional fluorescent nanomaterials like quantum dots and organic dyes are less photostable, more biotoxic, more environmentally sensitive, and prone to aggregation and contamination.

In other words, constrained by the generality of their physicochemical properties, the limitations of traditional nanomaterials in food heavy metal detection primarily stem from sensitivity issues caused by high background interference, insufficient selectivity, and cross-reactivity; potential toxicity and safety risks; and operational complexity affecting on-site applicability [10].

**Figure 1:**
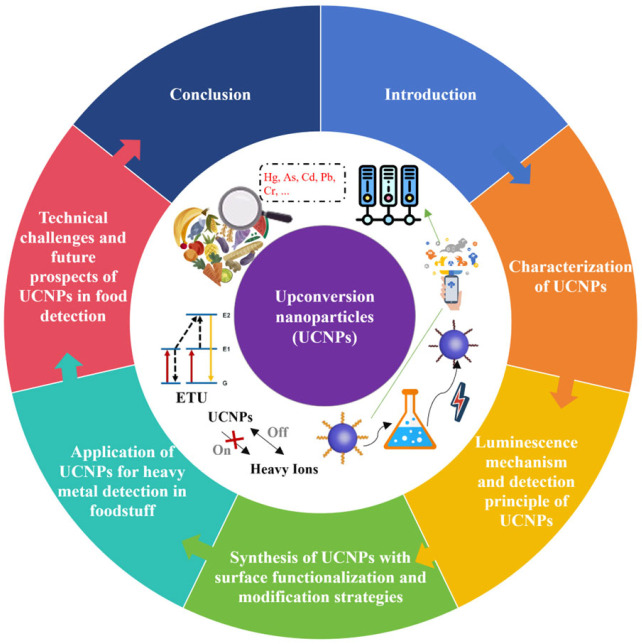
The logical structure of this review.

In recent years, fluorescence sensing technology based on upconversion nanoparticles (UCNPs) has become a research hotspot for heavy metal detection in food due to its unique optical properties, low environmental toxicity, and high chemical stability [11,12], and the optimization of its multi-modal functionality and biocompatibility further expands its application potentials in deep-tissue imaging and precise biologic detection. Focusing on the characteristics and detection advantages of UCNPs, this article reviews the detection principles and applications of UCNPs in food heavy metal detection, analyzes the technical challenges faced by UCNPs in practical applications, and looks forward to the future development of UCNPs in the field of food heavy metal detection (Figure 1).

## 2. Characterization of UCNPs and Their Advantages in the Detection of Heavy Metals

### 2.1. Characterization of UCNPs

Upconversion nanoparticles (UCNPs) are a class of inorganic nanocrystals doped with lanthanides, whose core property lies in their anti-Stokes luminescence, which is the emission of high-energy ultraviolet or visible photons through absorbing multiple low-energy near-infrared (NIR) photons and emitting high-energy UV or visible photons [13,14]. In other words, UCNPs are a class of nanomaterials with upconversion luminescence properties. This nonlinear optical process provides UCNPs with unique detection advantages. First, the NIR excitation light is usually 980 nm or 808 nm, which has lower energy and can significantly reduce the auto-fluorescence of biological samples, thus enhancing the detection signal-to-noise ratio [13,15,16]. Next, the NIR excitation light scatters and absorbs less in the biological tissues, providing it with the ability to penetrate deep tissues, and is suitable for in vivo imaging and in situ detection [16,17]. Furthermore, the UCNPs’ emission spectra have a narrow half-peak width (~20 nm), their luminescence lifetimes are as long as from microseconds to milliseconds, and background interference can be further eliminated by time-resolved techniques [14,18]. Compared with organic fluorescent dyes or quantum dots, UCNPs also exhibit excellent photo-stability and bio-compatibility, and their surfaces can be functionalized by SiO_2_ coating or ligand exchange, making them suitable for numerous biomedical applications [19,20,21].

### 2.2. Advantages of UCNPs for the Detection of Heavy Metals in Foodstuff

Based on the above characteristics, UCNPs show significant detection advantages in heavy metal detection in foodstuff. Their unique optical characteristics and functionalized designs provide efficient solutions for trace analyses in complex food matrices. High sensitivity and selectivity are one of the core advantages of UCNPs. Based on rare earth-element doping, UCNPs can realize ultra-sensitive detection through the unique luminescence mechanism of near-infrared excitation and visible-light emission. For example, UCNPs composited graphene oxide sensors can detect heavy metal concentrations down to the picomolar (pM) level through the change in fluorescence signals induced by the target substance (e.g., heavy metal ions or oligonucleotides) [22]. Further modification by metal–organic framework (MOF) coating or chiral nanostructures can significantly enhance the selective recognition of specific heavy metal ions like Pb^2+^, Hg^2+^ [23].

The ability to resist background interference is another key advantage of UCNPs in food detection. The near-infrared excitation light can effectively circumvent the auto-fluorescence interference of pigments, proteins, and other organic matter in the food matrix, thus substantially improving the signal-to-noise ratio [22,24]. This feature is particularly important in the detection of complex samples such as plant extracts and agricultural products [22]. To achieve portability and rapid detection, researchers have developed portable sensing systems based on smartphones. The optical signals of UCNPs can be directly compatible with cell phone cameras and combined with microfluidic chip or test strip technology so that the real-time analysis of heavy metals can be realized in the field of food processing without complex instruments [22,25].

The multifunctional integration potential of UCNPs further extends their application scenarios. Through the design of core–shell structures or composite nano-assemblies, signal amplification, selective adsorption, and optical response units can be integrated to enhance detection efficiency [23,26]. Such systems can also be combined with targeting molecules to enhance the specific recognition of heavy metals. In terms of detection performance, UCNPs-based sensors exhibit low detection limits with wide linear ranges. Through the fluorescence burst or enhancement effect, their detection limits can cover the nM–mM level, meeting the stringent requirements of food safety standards [22,27]. The environmental stability and reproducibility of UCNPs provide a guarantee for their practical applications. They have stable optical properties and strong resistance to photobleaching capacity. The stability of functionalized modified UCNPs materials in complex chemical environments has been significantly improved to ensure the reproducibility of detection results [28]. With high sensitivity, anti-interference capabilities, a portable design, and multi-functional integration capabilities, UCNPs provide an efficient and reliable technological platform for food heavy metal detection and have broad application prospects in the field of food safety detection.

## 3. Luminescence Mechanism of UCNPs and Its Detection Principle

### 3.1. Luminescence Mechanism of UCNPs

The anti-Stokes-shifted luminescence phenomenon of UCNPs promotes emission by converting NIR light to visible light or shorter wavelengths. In the determined food samples, the signal-to-noise ratio (SNR) of their fluorescence imaging is low because conventional fluorescent chromophores may also autonomously emit fluorescence. Unlike other fluorescent substances, such as visible spectroscopic probes and other conventional optical bioprobes, UCNPs can circumvent autofluorescent background interference during detection [29]. Therefore, the detection results obtained by fluorescence sensing technology based on UCNPs will be more accurate.

**Figure 2:**
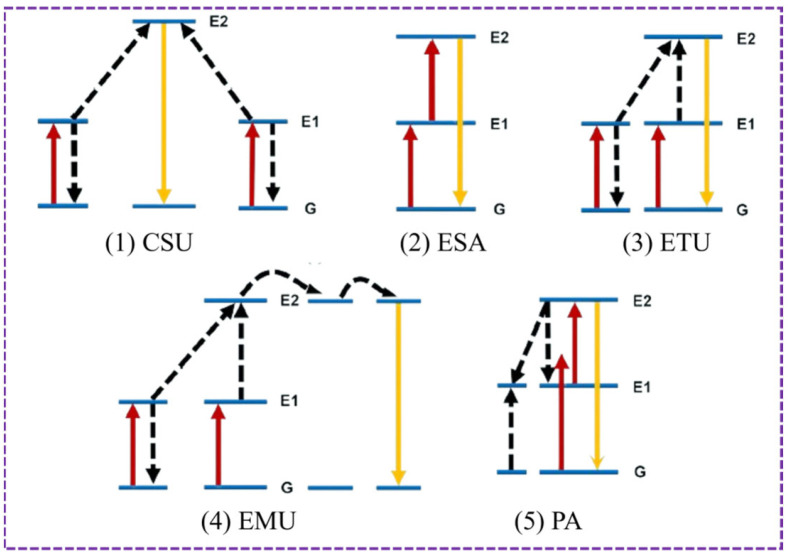
Luminescence mechanisms of UCNPs.

The luminescence of UCNPs is generally caused by the sequential absorption of two-photon or multi-photon particles, and the mechanisms include cooperative sensitization upconversion (CSU), excited state absorption (ESA), energy transfer upconversion (ETU), energy migration-mediated upconversion (EMU), and photon avalanche (PA) (Figure 2) [30]. CSU enhances energy transfer efficiency and boosts upconversion luminescence intensity through synergistic interactions between sensitizers and activators. ESA refers to the phenomenon that a single rare-earth ion sequentially absorbs low-energy photons, with electrons transitioning to an excited state before emitting high-energy photons to generate upconversion luminescence. After absorbing photons, the sensitizer transfers energy to adjacent activators. Activators transition to higher energy levels and emit high-energy photons, ETU, representing the most efficient upconversion mechanism at present. EMU achieves energy migration and efficient luminescence through a hierarchical design of sensitizers, energy accumulators, energy migrants, and activators within different layers of a core–shell structure. Under high excitation intensity, the synergistic interaction between ground-state absorption and cross-relaxation triggers avalanche-like energy accumulation, resulting in intense upconversion emissions. This is known as the PA mechanism.

The substrate, sensitizer, and activator together form the luminescent system of UCNPs. Among them, the matrix is the main structure of UCNPs, providing suitable lattice positions for the activator to form the energy level structure required for luminescence, while the sensitizer absorbs IR photon energy and transfers it to the activator, which completes the upconversion luminescence process in the low-loss environment provided by the matrix and decides the emission spectra according to the energy level structure of the activator ions, generating upconversion luminescence with different colors (for example, blue light emitted by Tm^3+^ and green light emitted by Er^3+^) [31].

### 3.2. Detection Principle of UCNPs in Food Heavy Metals

Regarding the design of sensors used in food analysis, the above luminescence mechanisms are helpful for designing relevant detection principles. Simply put, the five different mechanisms of UCNPs form the foundation for a novel and ideal detection platform, as well as scientific and rational interaction principles. For instance, when detecting food contaminants, like heavy metals, metalloids, and trace elements, UCNPs enable sensor construction through the following detection principles, based on one of those five luminescence mechanisms. This enhances sensitivity and selectivity, thereby enabling efficient monitoring of food safety.

**Figure 3:**
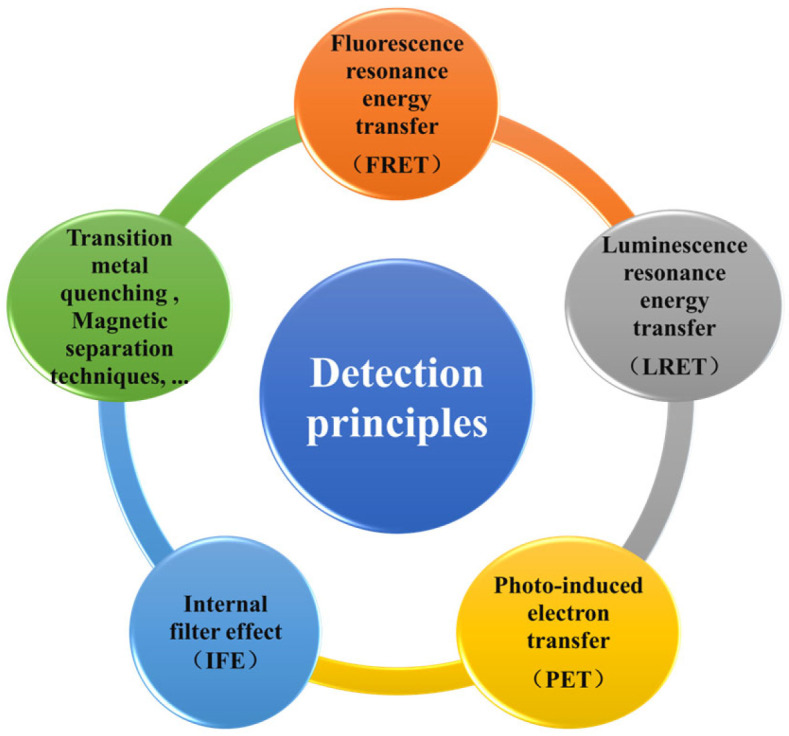
Principles of detecting heavy metals in foodstuff based on UCNPs.

In the actual application of UCNPs, the most common detection principle is the fluorescence quenching effect based on fluorescence resonance energy transfer (FRET). The FRET process generally involves nonradiative energy transfer through dipole–dipole interactions, which excites UCNPs to transfer energy to another fluorophore receptor, which leads to fluorescence quenching in UCNPs [32]. Other detection principles include luminescence resonance energy transfer (LRET), photo-induced electron transfer (PET), the internal filter effect (IFE), and transition metal quenching and magnetic separation techniques (Figure 3).

FRET principle: as energy donors, UCNPs have long fluorescence lifetimes, narrow band absorption and emission spectra, and highly efficient anti-Stokes luminescence. The detection principle of UCNPs for heavy metal detection in food is usually designed according to the FRET principle. It is important to note that such an effective FRET phenomenon occurs only when key conditions of energy transfer between the energy donor (i.e., UCNPs) and acceptor are met, including spectral overlap, distance limitation, and molecular orientation [33,34]. When the target substance binds with the energy receptor, the absorption spectrum of the latter may undergo a characteristic red-shift, blue-shift, or peak broadening, which directly affects the area of overlap with the emission spectra of UCNPs and thus regulates the efficiency of energy transfer. The specific binding between functionalized groups (e.g., carboxyl groups, amino groups, or aptamers) modified on the surface of UCNPs and the target substance can trigger a shortening of (<10 nm) or increase in the UCNP–receptor spacing. Some active substances can change the conformation of the ligands on the surface of UCNPs through reversible binding to form a dynamic energy transfer mode, leading to the ratiometric response of fluorescence signals. The spectral overlap effect is related to the distance of the ligands. For example, Hg^2+^ can induce a blue-shift in the absorption peak of the receptor and bring the UCNPs closer to the receptor through the binding of the thiol ligand, which can greatly enhance the sensitivity of the detection. Based on this, the heavy metal content in food can be accurately determined by quantitatively analyzing the change rule of the upconversion fluorescence intensity (ratio) of the specific emission peaks in UCNPs [35].

FRET is essentially a short-range, non-radiative energy transfer, strictly constrained by the donor–acceptor distance and sensitive to the surface structure of UCNPs. When the distance between UCNPs and energy receptors, such as gold nanoparticles and rhodamine derivatives is <10 nm, the energy is transferred to the receptor through the FRET principle and the fluorescence of UCNPs is quenched [36,37,38]. The presence of heavy metal analytes in food alters the interaction between the receptor and UCNPs, modulating the energy transfer efficiency through competitive binding, enzymatic reactions, or conformational changes. The fluorescence signal is thereby restored or altered for the quantitative detection of the target substance [37,38,39,40]. For example, the fluorescence of UCNPs is quenched by Au nanoparticles (AuNPs). However, Cu^2+^ can react with 4-mercaptobenzoic acid (4-MBA), a functionalized modifier on the surface of AuNPs, resulting in the separation of the AuNPs from the UCNPs, and the upconversion fluorescence of UCNPs is thus restored to reflect the Cu^2+^ concentration [41]. In addition, UCNPs, when used as donors, can avoid the photobleaching and auto-fluorescence interference of the traditional FRET system so as to enhance the sensitivity and specificity of detection [36,40].

LRET principle: The process of LRET principle is similar to that of the FRET principle, but the key difference lies primarily in the nature of the energy transfer mechanism and its distance dependence. LRET is a radiative energy transfer process that can tolerate greater distances and exhibits superior adaptability to steric hindrance. The LRET principle utilizes the upconversion luminescence through the near-infrared light excitation generated by UCNPs, and transfers the energy to the energy acceptor (e.g., organic dyes, gold nanoparticles) via nonradiative resonance. The excitation wavelengths of the LRET principle are located in the near-infrared region, so it can effectively avoid the interference of auto-fluorescence from biological samples and enhance the detection sensitivity [42,43,44]. Due to the larger anti-Stokes shift and higher photostability of UCNPs luminescence, they are more advantageous in deep-tissue in vivo imaging and complex biological environment detection [43,45].

PET principle: The PET principle refers to the process of electron transfer between an energy donor and energy acceptor, which can be subdivided into donor-excited PET (Donor-PET) and acceptor-excited PET (Acceptor-PET). Most of the PET principle based on UCNPs belongs to the former, where UCNPs regarded as energy donors transfer electrons to the acceptor. In more detail, the energy of the highest occupied molecular orbital (HOMO) of UCNPs is the lowest, but the energy of its lowest unoccupied molecular orbital (LUMO) is also between that of the energy acceptor’s HOMO and the energy acceptor’s LUMO. When the UCNPs are excited, their electrons jump from the HOMO to the LUMO, and then the electrons on the receptor’s HOMO are transferred to the HOMO of the UCNPs, resulting in the electrons of the UCNPs in the LUMO struggling to return to their HOMOs via the original pathway, which triggers a decrease in fluorescence or even fluorescence quenching [46]. For example, when the heavy metal ion Cu^2+^ binds to UCNPs, the PET process occurs within the system: the electron acceptor property of Cu^2+^ induces the transfer of excited state electrons of UCNPs to Cu^2+^ and results in a significant decrease in the fluorescence intensity of UCNPs. A linear relationship between the concentration of Cu^2+^ and the change in signals can be established by quantitatively detecting the extent of fluorescence quenching to achieve high-sensitivity detection [47].

IFE principle: IFE is also a fluorescence quenching phenomenon, the possibility of which depends on the overlap of light absorption between the energy donor (UCNPs) and the energy acceptor. When UCNPs emit upconversion luminescence (UCL) at a specific wavelength under near-infrared light excitation, if there is a target substance (e.g., heavy metal ions) in the system that can absorb the luminescence wavelength, the energy acceptor will directly attenuate the emitted light intensity of the UCNPs through light absorption, without the formation of a chemical bond or energy transfer [48]. In other words, the fluorescence signal is dynamically modulated by IFE to form a linear response when a change in absorption intensity is caused by varying concentrations of the energy receptor (target substance). Of course, IFE can be combined with other mechanisms (such as FRET) to form dual-mode or multi-mode detection. For example, cellulose-based sensors utilize IFE in synergy with intra-molecular charge transfer (ICT) to achieve the highly selective detection of Fe^3+^ [49,50]. Totally, IFE achieves signal modulation through spectral overlap, avoids the complex coupling steps of conventional fluorescent probes, and maintains high sensitivity and specificity while simplifying the design.

## 4. Synthesis of UCNPs with Surface Functionalization and Modification Strategies

### 4.1. Synthesis of UCNPs

As shown in Table 1, the synthetic methods of UCNPs mainly include the solvothermal method, thermal decomposition method, co-precipitation method, and biological template method. By controlling the reaction temperature and solvent system, solvothermal methods achieve size-controllable core–shell structure preparation and can improve biocompatibility through surface modification [51,52]. The thermal decomposition method uses precursors such as lanthanide oleates and has the advantages of high purity, a uniform particle size, and simplified purification steps, making it suitable for large-scale production [53]. The co-precipitation method modulates the luminescence properties and morphology by optimizing the fluorine source and surfactants, such as short-chain ligands, to enhance the water stability and fluorescence intensity [54,55]. Furthermore, the biological template method utilizes biomolecules (nucleotides) to introduce the synthesis and form a porous, environmentally friendly structure [56]. Surface modification techniques are the key to enhancing water dispersibility and bio-compatibility, and some of these methods can also achieve functionalization [57,58,59]. During the last decade, the development of high-throughput strategies has enabled the preparation of over 10 g of UCNPs in a single reaction, significantly improving the efficiency of synthesis [60].

### 4.2. Surface Functionalization Modification Strategies for UCNPs

**Figure 4:**
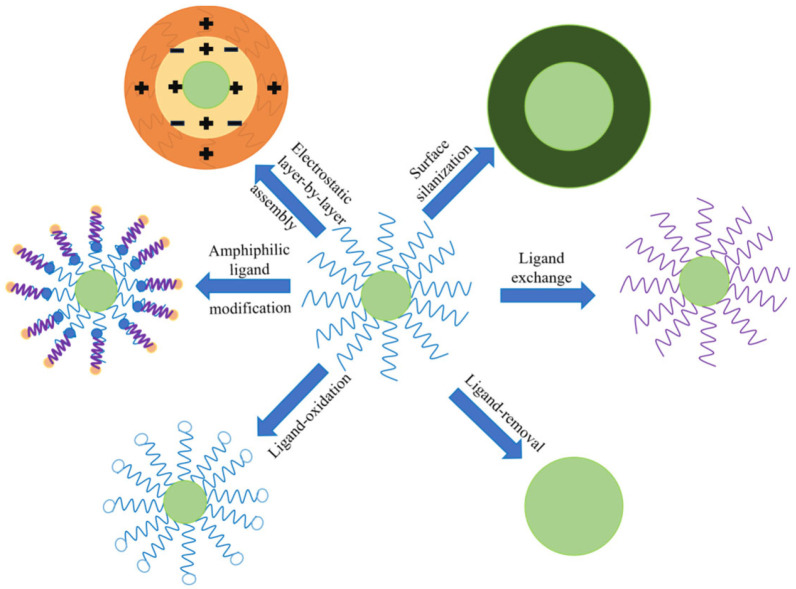
Strategies for the surface-functionalized modification of UCNPs.

In heavy metal detection in foodstuffs, UCNPs are usually hydrophobic on the surface after synthesis and struggle to directly disperse in the aqueous system or effectively bind to hydrophilic biomolecules; therefore, it is necessary to develop fluorescent probes based on UCNPs after surface functionalization modification to improve the colloidal stability and bio-compatibility of UCNPs in aqueous solution [65]. The functionalization strategies on the surface of UCNPs fluorescent probes are diverse (Figure 4) and mainly include the following categories according to usage frequency: (1) Ligand exchange strategy, which is the most widely used type of method. It utilizes strong adsorption groups, such as phosphoric acid, carboxylic acid, or sulfonic acid groups, to replace the original hydrophobic ligands. Phosphoric acid-based ligands can achieve a more complete ligand substitution due to their higher adsorption energy, enhancing aqueous dispersibility and stability [66]; (2) The surface silanization strategy, which is the modification of the surface of UCNPs through silane coupling agents to confer water dispersibility and biocompatibility, while retaining the hydrophobic end of the ligand to maintain colloidal stability [67]; (3) Amphiphilic ligand modification strategy, which involves anchoring amphiphilic polymers to the surface of UCNPs, with the hydrophobic end binding to the pristine ligand and the hydrophilic end being exposed to the aqueous phase, while providing the subsequent functionalization groups [68]; (4) Electrostatic layer-by-layer assembly strategy, which forms multilayer functional coatings by alternately adsorbing oppositely charged polyelectrolytes and it is suitable for drug-loaded and stimulus-responsive delivery systems [69,70]; (5) The ligand-removal strategy, which allows for the complete removal of surface ligands through high-temperature calcination or chemical treatment and introduces new hydrophilic layers, with the caveat that nanoparticle aggregation or structural damage must be avoided [71]; (6) The ligand-oxidation strategy, which converts the terminal double bonds of oleic acid or oleylamine ligands into carboxylic or amine groups through oxidation reaction to achieve hydrophilic modification; however, the conditions need to be optimized to prevent a decrease in colloidal stability [72].

## 5. Sample Pretreatment

Sample pretreatment involves steps such as sample conditioning, extraction, and digestion to eliminate matrix interference, enrich target heavy metal ions, or render them suitable for subsequent detection. The current sample pretreatment techniques emphasize the demand for efficient, non-toxic, low-cost, sustainable, and portable non-toxic sample preparation technologies. Sample pretreatment techniques are crucial in heavy metal detection in food, as they directly impact the accuracy, sensitivity, and environmental friendliness of the testing process.

Simple samples, namely common environmental samples like tap water or seawater, typically require minimal pretreatment—often just filtration through a membrane—but complex matrices necessitate additional techniques, such as microwave-assisted wet acid digestion, sugaring-out assisted extraction, or microfluidic device-based sample preparation.

Microwave-assisted wet acid digestion is an efficient sample digestion method for decomposing food matrices and releasing heavy metal ions, facilitating subsequent analysis. This approach enhances accuracy while reducing the time-consuming and complex issues associated with traditional digestion [73].

Sugaring-out assisted extraction is an innovative, environmentally friendly extraction technique specifically designed for heavy metal ions in dairy products like milk. It leverages the sugaring-out effect, employing acetonitrile as the extraction solvent to complete sample pretreatment without complex steps [74,75].

Microfluidic device-based sample preparation is a multi-detection sample pretreatment technology implemented on microfluidic platforms, encompassing cell capture and enrichment as well as nucleic acid sample preparation [76,77]. Although initially developed for foodborne pathogen detection, these preparation methods—such as capture and enrichment strategies—can be innovatively extended to heavy metal ion detection to reduce food matrix complexity and enhance sensitivity. Microfluidic approaches minimize traditional pretreatment steps, making them suitable for rapid point-of-care monitoring.

## 6. Application of UCNPs for Heavy Metal Detection in Foodstuff

Environmental pollution and serious diseases caused by heavy metals have become two major problems in global public health. Metals and metalloids, such as mercury (Hg), arsenic (As), cadmium (Cd), lead (Pb), chromium (Cr), and other heavy metals in the environment or living organisms, will accumulate in the body after entering the human body through the food chain, and long-term intake may lead to serious health risks, such as chronic poisoning, organ damage and even cancer [11,78]. The risk of heavy metal contamination is further exacerbated by the misuse of chemicals in the food production process and environmental pollution. Fortunately, as mentioned above, the new heavy metal detection technologies based on UCNPs can rapidly and accurately identify and detect trace heavy metal contaminants in foodstuff due to their high chemical stability, resistance to background interference, and low detection limit, so as to effectively protect food safety and human health. Consequently, the methods using UCNPs to solve the problems in the specific identification, trace level detection, and anti-interference analysis of heavy metals in complex food matrices according to different target element analytes are presented below (Table 2).

### 6.1. Detection of Mercury Ions

As the first of the “five poisons” of heavy metals, mercury (Hg) is highly toxic, and the bio-toxicity of its organic mercury (methylmercury, MeHg) ionic form is much greater than that of inorganic mercury (divalent mercury, Hg^2+^), which can be transmitted through the food chain and ultimately lead to bio-accumulation and toxicity amplification in the body, resulting in neurological damage and organ dysfunction, which is a serious threat to life and health because of its mutagenic, carcinogenic, and teratogenic effects [74]. Mercury ions are easily found in drinking water, aquatic products, agricultural products, and other foods, so there is an urgent need to develop specific and sensitive methods for the detection of mercury ions in different food matrices. The highly sensitive detection achieved by combining UCNPs with specific recognition elements and utilizing the FRET, LRET, or IFE principle of UCL is commonly adopted.

Annavaram et al. [80] developed fluorescent probes based on a hybrid system of rhodamine complex (RhDCPP) and UCNPs (NaYF_4_: Yb, Er), and showed that the RhDCPP-UCNPs fluorescent probes exhibited a significant enhancement of the selective absorption intensity in the presence of Hg^2+^. A significant spectral overlap between the emission and absorption band is observed, which leads to IFE-induced fluorescence quenching. The fluorescence quenching efficiency of the RhDCPP-UCNPs probe was proportional to the concentration of Hg^2+^, and the detection limit of Hg^2+^ in tap water could be as low as 13.5 nM, below the 30 nM limit standard set by the World Health Organization (WHO). Unlike the ANNAVARAM method, Wu et al. [81] proposed a dual FRET system using two colored UCNPs probes (NaYF_4_: Yb, Ho green UCNPs and NaYF_4_: Yb, Er red UCNPs) as the donor and AuNPs as the acceptor, regulating energy transfer through the matching of an aptamer and complementary DNA. In the presence of lead ions (Pb^2+^) and Hg^2+^, the aptamers preferred to bind with their respective analytes, forming a G-quadruplex structure for Pb^2+^ and a hairpin structure for Hg^2+^, and the dual FRET was thus disrupted, with green and red fluorescence restored. Among them, a linear relationship between fluorescence intensity and the logarithm of the Hg^2+^ concentration was observed in the Hg^2+^ concentration range of 0.5–500 nM, and the detection limit of Hg^2+^ was 150 pM, allowing for the detection of Hg^2+^ in aquatic products. Zhang et al. [82] made use of the UCNPs (NaYF_4_:Yb, Er@NaYF_4_) and the AuNPs to design a cysteine (Cys)-assisted anti-Stokes luminescence sensing platform (UCNPs-AuNPs), which was successfully applied to the detection of residual Hg^2+^ in green tea. AuNPs can effectively quench the luminescence of these UCNPs through the LRET process, while Cys can also interrupt the luminescence of the UCNPs by triggering the polymerization of AuNPs through the Au-S. However, due to the competitive effect of AuNPs on Cys, Hg^2+^ would weaken the efficiency of luminescence recovery, realizing the Hg^2+^ concentration-dependent luminescence change.

So far, various analytical techniques have been developed for Hg^2+^ and MeHg, but methods are based on UCNPs are rare due to the higher spatial site resistance and chemical inertness of MeHg, which requires particular modification on the surface of UCNPs for specific recognition. Liu et al. [83] prepared amphiphilic aqueous heptamethyl cyanine dyes (hCy7-UCNPs) modified by two long alkyl groups and a hydrophobic polymer (P-PEG), which were used as highly sensitive water-soluble probes for the UCL monitoring and bio-imaging of MeHg. Its optical titration experiments and upconversion luminescence live-cell imaging confirmed the superiority of hCy7-UCNPs in MeHg detection, with a detection limit as low as 0.18 ppb (far below the 0.8 ppm limit standard set by Codex Alimentarius Commission (CAC)) for MeHg using ratiometric upconversion luminescence as the detection signal. MeHg in vivo and in vitro were monitored by hCy7-UCNPs nanosystems via upconversion luminescence bio-imaging.

### 6.2. Detection of Arsenic Ions

Arsenic (As), a metalloid element, often exists in food in organic and inorganic forms. Inorganic arsenic ions are highly toxic and carcinogenic. The long-term intake of inorganic arsenic can cause skin lesions, cardiovascular diseases, and a variety of cancers, whereas organic arsenic ions have lower toxicity but may be metabolized to inorganic forms. Therefore, distinguishing and accurately detecting inorganic arsenic ions is crucial for food safety assessments and regulation, and is also a key monitoring indicator in food safety standards for various countries all around the world.

Xu et al. [84] synthetized multifunctional Fe_3_O_4_@NaGdF_4_:Yb:Er (Fe@UCNPs) with fluorescence and magnetic properties and developed LRET biosensors using aptamer-modified, magnetized Fe@UCNPs as the donors and tetramethylrhodamine (TAMRA) as the acceptors. Due to the highly integrated magnetic and fluorescent properties of Fe@UCNPs, inorganic arsenic As^3+^ in real aquatic products can be enriched by magnetic separation to avoid matrix interference while signal accumulation and amplification are achieved. Based on the LRET principle, Mondal et al. [85] created two luminescent nanoprobes through manganese dioxide (MnO_2_)-modified Er^3+^/Yb^3+^, with doped Ag_2_MoO_4_ UCNPs (cod-AMO-3/MnO_2_) as the energy donor and MnO_2_ as the energy acceptor. The UCL intensity of the cod-AMO-3/MnO_2_ fluorescent probe was significantly quenched by MnO_2_, but the presence of As^3+^ triggered the generation of a stable bi-nuclear diagonal bridging complex (As^5+^-MnO_2_), which led to gradual separation between the cod-AMO-3/MnO_2_ and MnO_2_. Subsequently, the UCL intensity was restored. The results showed that the linear range of detection of cod-AMO-3/MnO_2_ nanocomposites was 0–150 ppb, and the ultrasensitive detection limit of As^3+^ could be as low as 0.028 ppb, which was far below the 10 ppb standard limit in foodstuffs set by the WHO.

### 6.3. Detection of Cadmium Ions

Cadmium (Cd) is also a highly toxic heavy metal that can enter the food chain through environmental pollution or agricultural activities, and its long-term intake will lead to it accumulating in human kidneys and bones, leading to kidney function damage, osteoporosis, and even cancer. Therefore, the development of rapid and sensitive cadmium ion (Cd^2+^) detection technology is of great significance to safeguard food safety and human health.

Xu et al. [86] developed a dual-mode immunosensor based on UCL fluorescence and magnetic relaxation switch (MRS), in which UCNPs were combined with magnetic porous coordination network nanoparticles (Fe_3_O_4_@PCN-224) through the immunorecognition of antigens and antibodies to form a UCNPs/Fe_3_O_4_@PCN-224 assembly. The latter had good quenching ability and the fluorescence of UCNPs was thus quenched. The anti-background fluorescence interference property of UCNPs and the fast pre-fluidization ability of Fe_3_O_4_@PCN-224 in the magnetic field determined that the bio-sensing platform possessed a great advantage in solving the problem of interference with the fluorescence of pigments in opaque or colored samples, such as eggs, soya-bean milk, and red wine. However, the presence of Cd^2+^ affects the assembly process between UCNPs and Fe_3_O_4_@PCN-224; thus, when a UCL-MRS dual-mode immunosensor is used, Cd^2+^ in food can be detected, with detection limits of 0.038 ng/mL and 0.16 ng/mL, respectively. Bifunctional nanosensors based on small-molecule modulation have been widely used due to their simplicity, high sensitivity, and high selectivity. Sun et al. [87] designed a glutathione (GSH) calibrated bifunctional system based on the occurrence of FRET between UCNPs (NH_2_-NaYF_4_:Yb,Er/NaYF_4_@SiO_2_) and AuNPs for the detection of Cd^2+^ in drinking water. Unmodified AuNPs tended to aggregate in high-salt solutions and could easily quench the red fluorescence of UCNPs. Nevertheless, the presence of GSH prevented the aggregation of AuNPs and the red fluorescence of UCNPs was restored. However, Cd^2+^ can interact with GSH, which induced the aggregation of AuNPs, leading to the weakening of the red fluorescence of UCNPs. The detection limit of this system for Cd^2+^ was 0.059 μM.

Cadmium-tainted rice has emerged in an endless stream in the past two decades. Its root cause is that rice’s adsorption of Cd^2+^ is significantly stronger than that of other agricultural products; therefore, the determination of Cd^2+^ in rice is of more practical significance. Ding et al. [88] constructed a fluorescent sensor for 2-aminoethylphosphate dihydrogen sulfate (AEP) @UCNPs based on the IFE principle combined with the enzyme inhibition mechanism (AEP@UCNPs). It possessed stabilized fluorescence at 658 nm, which could be catalyzed by horseradish peroxidase (HRP) to oxidize tetramethylbenzidine (oxidized tetramethylbenzidine, HRP). AEP@UCNPs could be quenched by horseradish peroxidase (HRP)-catalyzed oxidized tetramethylbenzidine (oxTMB) via the IFE principle. When Cd^2+^ was present in the system, the enzymatic activity of HRP was inhibited, leading to a reduction in oxTMB and the recovery of AEP@UCNPs fluorescence. The detection of Cd^2+^ in rice could be realized within 30 min with a detection limit of 24.6 nM using the sensor, far below the 0.15 mg/kg limit standard set by the European Union (EU).

### 6.4. Detection of Lead Ions

Lead (Pb) is a known neurotoxin and carcinogen that can cause irreversible damage to the human body even at very low doses with prolonged exposure. The ingestion of lead may lead to impaired mental development, neurological dysfunction, anemia, renal impairment, and increased risk of cardiovascular disease and cancer in children. There is no safe threshold for the toxicity of lead, so the contents of lead ions in food need to be strictly controlled through testing.

Huang et al. [89] developed a DNAzyme-assembled and NIR light-excited nanosensor based on the LRET principle, which used UCNPs (NaYF_4_:Yb, Er) as the energy donor, DNAzyme-functionalized Black Hole Quencher 1 (BHQ1) as the energy acceptor, and the DNA enzyme as the target recognition motif for Pb^2+^. In the presence of Pb^2+^, the DNAzyme was activated and cleaved the substrate strand into two fragments at the RNA site, which led to substrate strand breakage and the restoration of luminescence. The sensor was also utilized for the successful application of highly sensitive and specific detection of Pb^2+^ in aquatic products. In contrast, the fluorescence sensor developed by Xu et al. [90] used carboxyl-modified UCNPs as the energy donor, gold nanorods (AuNRs) as the energy acceptor, and Cys as the bridge. The UCNPs-Cys-AuNRs sensor showed weak fluorescence signals, but the presence of Pb^2+^ could form a complex with Cys, restoring the UCNPs’ fluorescence. Under the optimal conditions, the relative fluorescence intensity of the sensor showed good linearity, with 1–100 μM Pb^2+^ and a detection limit of 0.5 μM (below the 20 μg/kg limit standard set by CAC), and was successfully applied to the detection of Pb^2+^ in matcha samples with recoveries in the range of 93.99–102.16% and relative standard deviations (RSDs) < 8%.

Electrochemical luminescence (ECL) sensors are widely used in Pb^2+^ detection, but their sensitivity needs to be improved. Gong et al. [91] designed a switching ECL sensor based on the resonance energy transfer (RET) principle between AuNPs and boron nitride quantum dots (BN QDs). The switching ECL sensor promoted intermediate SO_4_^¯^ generation through the reaction mechanism of Pb^2+^ recognition by DNA enzymes and substrate chain cleavage using poly(6-carboxyindole)/tin sulfide (P6ICA/SnS_2_) nanocomposites as a co-reaction gas pedal. Thanks to the combination of the DNA enzyme signal amplification strategy and the “on–off–on” switching design, the ECL sensor showed good selectivity, stability, and sensitivity, with a linear detection range of 10^−12^–10^−5^ M and a detection limit as low as 2.6 × 10^−4^ nM.

### 6.5. Detection of Other Common Trace Metal Ions

Chromium (Cr) mainly exists in nature as Cr^3+^ and Cr^6+^. The former is an essential trace metal ion involved in the synthesis of carbohydrates and lipid metabolism, but excessive intake may still lead to chromium deposition in tissues through the accumulation of the food chain and cause toxic effects, while the latter is highly toxic due to its strong oxidization, high solubility, and mobility, which may lead to high mutagenicity, carcinogenicity, and teratogenicity, such as DNA damage and organ toxicity. Therefore, precise detection of their speciation is needed to assess the safety risks, especially to prevent the long-term health hazards caused by the intake of Cr^6+^. Zeng et al. [92] reported a “off–on–off” fluorescent switch sensor based on citric acid-coated core–shell UCNPs (CA-UCNPs) and self-assembled copper porphyrin nanoparticles (nano-CuTPyP), which were detected by a switching fluorescent sensor. The nano 5, 10, 15, 20-tetrakis(4-pyridyl)-21H-23H-porphine copper obtained by the acid-base neutralization micellar confinement self-assembly method has excellent optical properties and water-solubility, and can be used as an effective quencher. In addition, positively charged nano-CuTPyP was attracted to the surface of negatively charged CA-UCNPs to quench their fluorescence through electrostatic interaction. In the presence of Cr^6+^, nano-CuTPyP can react with Cr^6+^ to form a nano-CuTPyP/Cr^6+^ complex, which separated nano-CuTPyP from CA-UCNPs, and the fluorescence of the latter when excited at 808 nm was restored. The sensor showed good linear response to 0.5–400 µM Cr^6+^, with a detection limit of 0.36 µM (below the 2.0 mg/kg limit standard according to GB 2762-2025 National Food Safety Standard: Maximum Contaminant Levels in Foods set by China), and was successfully applied to the sensitive and specific detection of Cr^6+^ in agricultural and aquatic products.

As an essential nutrient trace element, the content of iron (Fe) directly affects hemoglobin synthesis, energy metabolism, and immune function. Excessive intake may lead to acute toxicity or chronic organ damage, especially the misuse or abuse of iron supplements, which may cause serious health risks. The detection of Fe content in foods can ensure compliance with nutritional fortification standards, avoiding the decline in the quality of life of patients with anemia or chronic diseases due to deficiency, as well as preventing safety hazards caused by industrial contamination or abnormalities in iron morphology during processing. Zhang et al. [93] synthesized a novel Schiff base-functionalized cyano derivative (CyPy), which was successfully assembled on the surface of UCNPs via amphiphilic polymer encapsulation. The UCNP contained the Fe^3+^-recognizer CyPy as the energy donor and UCNPs (*β*-NaYF_4_:Yb, Er) as the energy acceptor. The efficient energy transfer from CyPy to *β*-NaYF_4_:Yb, Er endowed the nanofluorescent probe with high sensitivity to Fe^3+^ in water, and its detection limit was as low as 0.21 μM, (below the 0.3 mg/L limit standard set by China). This was successfully applied to the determination of Fe^3+^ in drinking water, with RSD recoveries of 97–106% and <3.72%.

Copper (Cu) is a key cofactor for many enzymes in human metabolism, but excessive intake can lead to liver damage, neurological dysfunction, and other health risks. Food safety needs to be assessed through determination in cases where environmental factors may lead to excessive levels of copper in food, where copper contamination may be introduced during food processing, and where toxic effects may be exacerbated by the coexistence of multiple heavy metals in complex matrices. Therefore, copper ion (Cu^2+^) detection is important to ensure the nutritional safety of food so as to prevent chronic poisoning and to assess the impact of environmental pollution. Su et al. [94] demonstrated a highly sensitive and selective NIR-excitable poly(acrylic acid) (PAA)-coated sensor based on UCNPs, and constructed the PAA-modified Na(Yb, Nd)F_4_@Na(Yb, Gd)F_4_:Tm@NaGdF_4_ core–shell structure UCNPs sensor via the co-precipitation route. It was shown that Cu^2+^ could react with PAA-UCNPs to form a copper carboxylate complex, leading to the weakening or even quenching of the latter’s fluorescence. The results showed that the PAA-UCNPs sensor had a high selectivity for Cu^2+^ with a detection limit of 0.1 μM. The development of single-nanoparticle-level sensing technology has further enhanced the sensitivity of Cu^2+^ detection. Wang et al. [95] introduced a single-nanoparticle-level nanosensor utilizing yttrium sodium fluoride (NaYF_4_) doped with ytterbium (Yb^3+^) and erbium (Er^3+^) UCNPs as an energy donor with a black hole quencher 1 (BHQ1) dye attached to its surface and a Cu^2+^-dependent DNA enzyme as an energy acceptor. When a certain amount of Cu^2+^ was added, the BHQ1-containing molecules in the DNA enzyme were cleaved, resulting in the restoration of the fluorescence that was originally quenched. The results showed that the sensor had SNRs (>277) of three orders of magnitude (from sub-nM to μM) at all measured concentrations, with detection limits as low as 220 pM, below the 10 mg/kg limit standard set by China.

## 7. Technical Challenges and Future Prospects of UCNPs in Food Detection

### 7.1. Current Challenges

As mentioned above, UCNPs possess numerous advantages. Compared with other novel detection methods, such as those based on microneedles, quantum dots, metal–organic frameworks, or carbon dots, however, they exhibit a series of drawbacks in practical applications, including but not limited to low brightness or luminous intensity, low quantum efficiency and energy conversion efficiency, surface quenching effects, instability and degradation in aqueous media, long radiative lifetimes, thermal quenching issues, weak emission under low-power excitation, background signal interference, poor physiological stability and lack of effective targeting, high variability in cellular uptake results, lack of standardization in surface functionalization, limited applications, and absence of unified standards [96,97,98,99,100,101,102,103,104,105,106,107].

Then limited toxicological and environmental risk data may pose potential safety concerns [108,109], particularly as the ecotoxicity pathways generated during lanthanide doping remain unknown. Furthermore, UCNPs may dissolve or quench during processing or degradation, creating long-term environmental hazards [100]. This necessitates disposal management strategies. Existing research has insufficiently addressed waste treatment approaches or regulatory frameworks, potentially creating regulatory gaps. Systematic data acquisition and evaluations are urgently needed to guide safe application and environmental policy development.

Especially in the field of heavy metal detection for foodstuff, the applied technologies based on UCNPs still face multiple challenges. First, lipids and proteins in complex food matrices are easily and non-specifically adsorbed on the surface of UCNPs, leading to interference in the detection signal or inactivation of the probe. This requires the optimization of the sample pretreatment process, which can be achieved by magnetic separation or enzymatic purification or the development of anti-fouling coatings (e.g., polyethylene glycol modification) to enhance the stability of the probe. Secondly, there are technical bottlenecks in the probe design for simultaneous multi-target detection, such as the synthesis of UCNPs at different wavelengths needs to balance the efficiency and stability of luminescence. However, the crosstalk problem between multiple signal channels (cross-response of FRET, etc.) limits the detection accuracy. Moreover, the coverage of food analysis based on UCNPs is insufficient. Currently, this mainly focuses on common toxic and hazardous heavy metals, while little research has been conducted on monitoring the excessive intake of essential nutrient trace metal elements, such as zinc (Zn). Additionally, the morphological diversity of heavy metals in food (e.g., inorganic vs. organic mercury, inorganic vs. organic arsenic, Cr^3+^ vs. Cr^6+^) requires the probes to have the ability of morphology-specific recognition, which poses a greater challenge to the design of ligands for the functionalization of the surface modification of UCNPs.

### 7.2. Future Prospects

The rapid development of big data and artificial intelligence (AI) brings new hopes and possibilities for guaranteeing food safety. Wang et al. [110] first introduced the concept of transfer learning into the quantitative detection of UCNPs. They designed an AI-based solution strategy for the quantitative detection of UCNP-lateral flow assays (LFAs) in small datasets without special preprocessing. By deploying the trained transfer learning model in a local IoT device, an efficient, universal, portable commercial internet of things (IoT) device for upconversion luminescence quantitative detection was developed, which can infer highly accurate results in real-time in only 20 s. Li et al. [111] also established a smartphone-based colorimetric method by capturing the changes in the fluorescence colors of probes exposed to different Hg^2+^ concentrations. They designed a dual-emission ratiometric fluorescence probe based on Zr-based metal–organic frameworks (Zr-MOF) and Ag nanoclusters (AgNCs) for the sensitive and visual detection of Hg^2+^ in the Porphyra matrix and achieved analytical recoveries of 94.74–101.1%, indicating the practicability of the method.

Looking ahead, the development of heavy metal detection technologies in foodstuff based on UCNPs holds great promise in the field of technological innovation and scenario expansion. On the one hand, through the development of multimodal integrated sensors, the fluorescence characteristics of UCNPs can be combined with electrochemical detection, surface-enhanced Raman spectroscopy, or microfluidic chip technology, which can break through the limitations of a single optical signal and realize the multi-dimensional analysis of heavy metal speciation and concentration. On the other hand, through using the near-infrared excitation properties of UCNPs, the potential of real-time dynamic monitoring in the food processing chain can be explored, for example, by embedding functionalized UCNPs into food packaging materials to track the migration pathway or accumulation behavior of heavy metals in real time. In addition, miniaturized portable devices (e.g., the development of smartphone-enabled detection platform apps) and automated data-processing algorithms will be designed to accelerate the transition of UCNPs technology from the laboratory to rapid detection in the field. Through bio-mimetic material design and bio-mimetic interface engineering, this is expected to further expand the application of UCNPs in living food models of heavy metal metabolism, such as plants or other organisms.

## 8. Conclusions

Due to their unique optical properties, good bio-compatibility, and tunable interfacial chemistry, UCNPs have revealed significant detection advantages for multi-ion analysis, such as heavy metal ions, trace metal ions and metalloid ions, in food matrices. Understanding the properties, luminescence mechanism, synthesis methods, and functionalization surface modification strategies of UCNPs further helps us to focus on the detection principle of UCNPs through these novel methods, and allowed for the application of UCNPs in the detection of heavy metal ions, trace metal ions and metalloid ions in complex food matrices in recent years.

Nevertheless, compared with other novel detection methods, such as those based on microneedles, quantum dots, metal–organic frameworks, or carbon dots, some challenges regarding UCNPs are still present in practical applications, such as low brightness or luminous intensity, low quantum efficiency and energy conversion efficiency, and surface quenching effects. The continuous optimization and improvement of their design are the main measures to enhance performance. With the development of new technologies, novel detection methods based on UCNPs also should be developed that can integrate fully with AI, portable smart devices, or Big Data. The widespread application of UCNPs in food safety detection still has great potential for development.

## Figures and Tables

**Table 1:** Methods of the synthetic preparation of UCNPs.

Methods	Strengths	Weaknesses	Ref.
Solvothermal method	Cheap raw materials; easy and effective operation; adjustable particle size, crystal phase, and morphology; relatively low reaction temperature; no requirement for high-temperature post-heat treatment	Difficult to screen for optimal synthesis conditions; requires specific reaction vessels; product size is generally large	[51,52]
Thermal decomposition method	The prepared product is highly crystalline, pure, and homogeneous, with good nanocrystalline morphology	The reaction requires high temperatures and anaerobic and anhydrous environments; the generation of toxic by-products and non-polar capping ligands limits its further application in food determination	[53,61,62]
Co-precipitation method	Does not require expensive instrumentation, stringent reaction conditions, and complex operating procedures; the synthesized products have high yields and fast growth rates	Poor product shape; uneven particle size; high temperature heat treatment required to obtain the product	[54,55,63]
Biological template method	Gentle and environmentally friendly; utilizes the natural structure of biomolecules to precisely regulate particle size, morphology, and bio-compatibility	Complex template removal; low yield; high temperature or extreme reaction conditions may destroy the template structure; residual biomolecules may affect the optical properties of the product, making it difficult to produce on a large scale	[56,64]

**Table 2:** Types of heavy metals detected in food based on UCNPs.

Types	Target Elements	Main Species	Types of Food	Detection Principles	Limit of Detection by the Developed Method	Recovery (%)	Ref.
Toxic harmful heavy metals	Hg	Hg^2+^, MeHg	Tea, tap water	LRET, IFE, FRET	0.15–13.5 nM	97.20–112.00	[79,80,81,82,83]
	As	As^3+^, As^5+^	aquatic products	LRET	0.028 nM	94.34–103.34	[84,85]
	Cd	Cd^2+^	egg, soymilk	FRET	0.038–59.0 nM	97.30–109.78	[86,87,88]
Trace elements for the human body	Pb	Pb^2+^	Zebrafish, matcha, water	LRET, FRET	2.6 × 10^−4^–500.0 nM	93.99–102.16	[89,90,91]
	Cr	Cr^3+^, Cr^6+^	Rice, fish	FRET	360 nM	92.00–108.20	[92]
	Fe	Fe^3+^	Tap water	LRET	210 nM	95.00–106.00	[93]
	Cu	Cu^2+^	Environmental water	FRET	0.22–100 nM	Not mentioned	[94,95]

## Data Availability

No new data were created or analyzed in this study.

## Abbreviations

The following abbreviations are used in this manuscript:

UCNPs	Upconversion Nanoparticles
NIR	Near-Infrared
MOF	Metal–Organic Framework
CSU	Cooperative Sensitization Upconversion
ESA	Excited State Absorption
ETU	Energy Transfer Upconversion
EMU	Energy Migration-mediated Upconversion
PA	Photon Avalanche
FRET	Fluorescence Resonance Energy Transfer
LRET	Luminescence Resonance Energy Transfer
PET	Photo-induced Electron Transfer
IFE	Internal Filter Effect
AuNPs	Au Nanoparticles
HOMO	Highest Occupied Molecular Orbital
LUMO	Lowest Unoccupied Molecular Orbital
UCL	Upconversion Luminescence
ICT	Intra-molecular Charge Transfer
LOD	Limit of Detection
WHO	World Health Organization
CAC	Codex Alimentarius Commission
MRS	Magnetic Relaxation Switch
HRP	Horseradish Peroxidase
TMB	Tetramethylbenzidine
EU	European Union
BHQ	Black Hole Quencher
RSD	Relative Standard Deviation
ECL	Electrochemical Luminescence
RET	Resonance Energy Transfer
BN	Boron Nitride
QD	Quantum Dot
PAA	Poly(Acrylic Acid)
SNR	Signal-to-Noise Ratio
AI	Artificial Intelligence
LFA	Lateral Flow Assay
AgNCs	Ag Nanoclusters
